# Clonally Diverse Methicillin and Multidrug Resistant Coagulase Negative Staphylococci Are Ubiquitous and Pose Transfer Ability Between Pets and Their Owners

**DOI:** 10.3389/fmicb.2019.00485

**Published:** 2019-03-26

**Authors:** Elena Gómez-Sanz, Sara Ceballos, Laura Ruiz-Ripa, Myriam Zarazaga, Carmen Torres

**Affiliations:** ^1^Institute of Food, Nutrition and Health, ETH Zürich, Zurich, Switzerland; ^2^Área de Microbiología Molecular, Centro de Investigación Biomédica de La Rioja (CIBIR), Logroño, Spain; ^3^Área Bioquímica y Biología Molecular, Universidad de La Rioja, Logroño, Spain

**Keywords:** methicillin-resistant coagulase negative staphylococci, *Staphylococcus epidermidis*, multidrug resistance, interspecies transmission, carriage dynamics, co-carriage, owner, pet

## Abstract

Sixty-eight owners and 66 pets, from 43 unrelated pet-owning households were screened for methicillin-resistant coagulase negative staphylococci (MRCoNS), potential cases of MRCoNS interspecies transmission (IT), and persistence. MRCoNS isolates were identified by microbiological and molecular tests. MLST-based phylogenetic analysis was performed in *Staphylococcus epidermidis* isolates. Antimicrobial susceptibility was evaluated using phenotypic and molecular methods. SCC*mec* type and the presence of biofilm-related *ica* locus was PCR-tested. Isolates suspected for MRCoNS IT cases were subjected to *SmaI*-PFGE analysis and individuals from positive households were followed-up for 1 year for carriage dynamics (every 3 months, T0–T4). Nineteen MRCoNS isolates from owners (27.9%) and 12 from pets (16.7%) were detected, coming from 20 households (46.5%). *S. epidermidis* was predominant (90 and 67% of human and animal strains, respectively), showing high phylogenetic diversity (16 STs among 24 strains). Methicillin-resistant *S. epidermidis* (MRSE) strains belonged to CC5 (75%), CC11 (12.5%), singleton S556 (8.3%), and S560 (4.17%). Significant host-associated differences were observed for resistance to aminoglycosides, co-trimoxazole, chloramphenicol (higher in animal isolates) and tetracycline (higher among human strains). Multidrug resistance (MDR) was common (68.4%) and associated with human strains. Great diversity of *ccr* and *mec* complexes were detected, most strains being non-typeable, followed by SCC*mec*IV and V. Over one third of isolates (most from owners), carried the *ica* locus, all MRSE CC5. Two sporadic IT cases (T0) were identified in owners and dogs from two households (4.7%), with diverse interspecies-exchanged clones detected along the sampling year, especially in dogs. A comparative analysis of all MRCoNS, with all nasal coagulase positive staphylococci (CoPS) recovered from the same individuals at T0, revealed that CoPS alone was predominant in owners and pets, followed by co-carriage of CoPS and MRCoNS in owners but single MRCoNS in pets. Statistical analyses revealed that owners are more prone to co-carriage and that co-existence of IT cases and co-carriage are positively interrelated. MRCoNS from healthy owners and their pets are genetically heterogeneous MDR strains that are spread in the community. Therefore, pets also contribute to the dissemination of successful human clones. Owner-pet inhabitancy increases the risk for staphylococcal temporal concomitance with its subsequent risk for bacterial infection and genetic exchange.

## Introduction

Staphylococci are normal commensal bacteria of the skin and mucous membranes of humans and other animals. They can be differentiated by their ability to produce coagulase. Coagulase positive staphylococci (CoPS), with *Staphylococcus aureus* as major representative in humans and *Staphylococcus pseudintermedius* in dogs, pose, in general, higher pathogenic potential than coagulase negative staphylococcal (CoNS) species (Becker et al., 2014). CoNS are less often involved in community-associated diseases, but represent one of the major nosocomial pathogens, and have a substantial impact on human life and health (Becker et al., 2014; May et al., 2014). In humans, *Staphylococcus epidermidis* is the most common species among CoNS infections (24–80%), and the most frequent cause of medical device-associated infections (Miragaia et al., 2009; Becker et al., 2014). Regardless of the sparse data available, CoNS have occasionally been confirmed as causative agents for different site infections in dogs (Malik et al., 2006; Kern and Perreten, 2013; LoPinto et al., 2015; Couto et al., 2016). Yet, their zoonotic potential and importance in veterinary medicine is unclear.

Staphylococci, especially CoNS, are notorious for their ability to accrue antimicrobial resistance (AMR) determinants and to produce a biofilm, which makes associated infections particularly difficult to treat (Miragaia et al., 2009; Becker et al., 2014). Further, methicillin resistance is normally associated with additional resistances, which may pose a risk for the AMR gene transfer between staphylococci with higher pathogenic properties, such as *S. aureus* (Bloemendaal et al., 2010). On top of this, multidrug resistant (MDR) strains drastically limit the therapeutic options available and represent a human and animal health problem.

Nasal *S. aureus* and *S. pseudintermedius* can be exchanged between owners and cohabitant pets, and such acquisition can persist over time (Gomez-Sanz et al., 2013a,b). However, no data are available on the incidence and diversity of MRCoNS in healthy owners and their companion animals at the household, on potential cases of interspecies transmission (IT) and on its persistence over time.

The potential association between owner-pet companionship and the concomitant carriage of more than one staphylococcal type (CoPS and MRCoNS), as well as the potential host tropism for these subpopulations is unknown, but is essential to appraise potential owner-pet cohabitation as a risk factor for staphylococcal acquisition, infection and transmission. In addition, simultaneous carriage of CoPS and MRCoNS represents a potential risk for AMR transfer, which is barely considered in AMR surveillance studies.

The **goal** of this study is to determine the nasal occurrence, diversity, clonal distribution, and molecular characterization of MRCoNS in healthy owners and their pets, residing in common households, as well as to address potential IT cases and their carriage dynamics. We subsequently analyzed the MRCoNS and concomitant CoPS nasal patterns to determine whether there was any bacterial species- and/or host-associated tropism.

## Materials and Methods

### Study Population and Sampling Criteria

Individuals from 43 unrelated pet-owning households were sampled in La Rioja region (Northern Spain) for the nasal carriage of MRCoNS and for IT potential cases. IT was defined as the presence of the same MRCoNS clone in owner and cohabitant pet. Samples were taken from March 2009 to February 2011. Individuals tested were, in parallel, sampled for the nasal occurrence of CoPS (Gomez-Sanz et al., 2013b). Only MRCoNS were further characterized in this study. Inclusion criteria for households tested included healthy humans whose profession did not involve any direct animal contact. None of the individuals tested had received antimicrobial treatment within the 4 months prior sampling. Household recruitment was on a voluntary basis. Sixty-eight humans and 66 animals (54 dogs, 12 cats) were included (Gomez-Sanz et al., 2013a,b). All individuals gave written informed consent to participate in this study, as well as for the sampling of their animals. This study was included in a project approved by the Ethical Committee of Clinical Research of La Rioja (reference: METC 09-399/C). One to five owners and one to five pets were tested from each household, showing 10 different combinations. In most cases (19, 44.2%), only one person and one animal were sampled per household. Nine and 11 of the 43 household units included more than one pet (20.9%) and more than one owner (25.6%), respectively. Four households included both more than one animal and more than one owner (9.3%). In total, 36 of 66 pets lived with other sampled animals (dog/cat) (54.5%), while 40 of 68 owners lived with other sampled humans (58.8%). Of note, all cohabitant pets within a sampled household were included in the study whereas owners were not always all sampled. Swabs were transported to the lab within 5 h after sampling and were either immediately analyzed or stored at -20°C until further analysis.

### Isolation and Identification of MRCoNS

Sampled nasal swabs were inoculated into Brain-Heart-Infusion broth (BHI, Difco) supplemented with 6.5% NaCl and incubated at 37°C for 24 h. One-hundred microliters were inoculated on Oxacillin-Resistant-Staphylococcal-Agar-Base (ORSAB; OXOID) plates supplemented with 2 mg/L of oxacillin. Plates were incubated at 35°C for 24–48 h. All blueish to white (potential MRCoNS) colonies with different morphologies were sub-cultured on BHI agar and further studied. Preliminary identification of MRCoNS isolates was based on colony morphology, Gram staining, and catalase and DNase activities. Presence of the *mecA* gene was investigated by PCR in all isolates (Gomez-Sanz et al., 2013a). Identification of MRCoNS was performed by amplification and sequencing of the *sodA* gene in all *mecA* positive CoNS isolates (Poyart et al., 2001). In addition, isolates that were difficult to type by Multi Locus Sequence Typing (MLST) were also identified by amplification and sequencing of the 16S rRNA (Hogg and Lehane, 1999), and by matrix assisted laser desorption ionization-time of flight mass spectrometry (MALDI-TOF MS). When different isolates from the same individual were recovered, which belonged to the same bacterial species and shared the same AMR phenotype, only one isolate was further characterized. Individual nomenclature was as follows: household number (1–43) – isolate host [Human (H); dog (D); cat (C)]. – number of individuals when more than one (1–5).

### Multi-locus Sequence Typing (MLST) of Methicillin Resistant *S. epidermidis* (MRSE) Isolates

All 25 MRSE isolates were subjected to MLST as recommended by Thomas et al. (2007). Two novel sets of primers for *aroE* (aroE-fw2: 5′-TTCATTATCGCATTGATGC-3′, aroE-rv2: 5′-TCAGCACCTTGATGAACGAA-3′) and *tpi* (tpi-fw2: 5′-TAGCCGGAAACTGGAAAATG-3′, tpi-rv2: 5′-GCACCTTCTAACAATTGTACG-3′) alleles were employed for isolates that could not be amplified with the standard primers. Allele and ST identification was used following the *S. epidermidis* MLST database^1^. The MLST data were analyzed using the goeBURST algorithm^2^ for ST clustering within clonal complexes (CC) (as of November 2017). For this, Phyloviz2 grouping was generated by Hierarchical Clustering (Hamming Method, UPGMA) using allelic profiles (Nascimento et al., 2017). In addition, a phylogenetic relationship of concatenated sequences was investigated by the construction of a distance tree including metadata on isolates characteristics for each of the different MLST profiles obtained (CLC Genomics Workbench 10.0.1, Qiagen Bioinformatics).

### Staphylococcal Cassette Chromosome *mec* (SCC*mec*) Classification

The SCC*mec* type was determined based on the chromosomal cassette recombinase *ccr* gene/s and on the type of *mec* complex as described by Kondo et al. (2007), while confirmation of SCC*mec* type was tested using SCC*mec* primers described by Zhang et al. (2005). In addition, allele *ccrAB4*, present in SCC*mec* types VI and VIII (Oliveira et al., 2006) was included. Following this approach, cassettes I–IX could be identified.

Typeability of the SCC*mec* cassettes was defined as follows: (i) Typeable (T) SCC*mec* cassettes were considered those for which *ccr*, type of *mec* complex (Kondo et al., 2007) and/or SCC*mec* (Zhang et al., 2005) were identified; (ii) Non-Ascribed (NA) SCC*mec* types were those with a novel combination of *ccr*, *mec* complex, and/or SCC*mec*, and (iii) Non-Typeable (NT) were considered those that did not yield positive results with the primer sets used, per scheme. New SCC*mec* were defined as those enclosed within NA and NT categories.

### Characterization of Antimicrobial Resistance Profile

Susceptibility to 17 antimicrobial agents was performed using an agar disk-diffusion method (CLSI, 2013). Antimicrobial agents tested were as follows (class of agent/s): penicillin, oxacillin [+ 2% NaCl], cefoxitin (β-lactams); gentamicin, kanamycin, tobramycin, streptomycin (aminoglycosides); co-trimoxazole (aminopyrimidine/sulfonamide); erythromycin (macrolides); clindamycin (lincosamides); tetracycline (tetracyclines); chloramphenicol (amphenicols); vancomycin (glycopeptides); ciprofloxacin (fluoroquinolones); mupirocin (pseudomonic acid); fusidic acid (steroids); and linezolid (oxazolidinones). Procedures and breakpoints were those proposed for CoNS in CLSI document M100-S23 (CLSI, 2013). For streptomycin and fusidic acid, the methods and breakpoints employed were those recommended by the Société Française de Microbiologie^3^. The double-disk diffusion test (D-test) was performed on all isolates to detect inducible clindamycin resistance (CLSI, 2013). Multidrug resistance (MDR) was considered when a resistance to > 3 antimicrobial classes was observed.

The presence of 33 AMR genes, in addition to the *mecA* gene, was investigated by PCR: *blaZ*, *tet*(K), *tet*(M), *tet*(L), *erm*(A), *erm*(B), *erm*(C), *erm*(T), *erm*(F), *mph*(C), *msr*(A)/*msr*(B), *lnu*(A), *vga*(A), *vga*(C), *aacA-aphD, aphA3, aadE*, *aadD*, *aadA, str*, *dfr*(A), *dfr*(D), *dfr*(G), *dfr*(K), *mupA*, *fexA, cfr, cat_pC194_, cat_pC221_, cat_pC223,_ fusB*, and *fusC* (Gomez-Sanz et al., 2013a,b). Positive controls from the collection of the University of La Rioja were included in each reaction.

Mutations within the quinolone resistance determining region (QRDR) of *gyrA* and *gyrB* genes (DNA gyrase subunits), and within *parC* and *parE* genes (DNA topoisomerase IV subunits) were investigated in ciprofloxacin resistant isolates (Yamada et al., 2008). The corresponding genes of the quinolone susceptible *S. epidermidis* strain ATCC 12228 (GenBank ac. no NZ_CP022247.1) were used as a reference for mutation detection and positioning within the gene.

### Presence of Virulence Genes Involved in Biofilm Formation

PCR based determination of several genes involved in biofilm formation was implemented. Genes tested were the *S. aureus* biofilm matrix protein *bap* (Cucarella et al., 2001); the Staphylococcal intercellular adhesin (*icaADBC*) operon-containing genes *icaA*, *icaB*, *icaC*, and *icaD*, responsible for the synthesis of the biofilm matrix polysaccharide intercellular adhesion (PIA) (Ziebuhr et al., 1999; Arciola et al., 2006); the transcriptional repressor of the *ica* locus, the *icaR* gene (Conlon et al., 2002); as well as the insertion sequence IS*256*, which has been observed to play a role in phase variation of virulence by *ica* locus in *S. epidermidis* (Ziebuhr et al., 1999).

### Determination of Cases of Interspecies Transmission (IT)

The genetic relatedness of MRCoNS isolates suspected for cases of direct IT – i.e., those isolates of the same species recovered from cohabiting individuals that exhibited identical AMR profile, MLST for *S. epidermidis*, and SCC*mec* type – was addressed by Pulsed Field Gel Electrophoresis (PFGE) of the total DNA digested with a *SmaI* macro-restriction enzyme following the HARMONY protocol (Murchan et al., 2003).

### Longitudinal Approach: Carriage Status Definition and IT Dynamics

All individuals from households with cases of direct IT were followed-up with for a year. For this, nasal samples from the anterior nares of owners and pets were studied once every 3 months (five sampling times in total, T0–T4) with a total of 24 additional samples analyzed (T0–T4). Studied subjects positive for MRCoNS in at least four of the five samplings (including T0) were considered persistent carriers; those positive in two or three samplings were defined as intermittent carriers; individuals positive in a single sampling were reported sporadic carriers; and those negative throughout the study were defined as non-carriers. Dynamics of the IT cases over time was defined likewise (persistent, intermittent, and sporadic).

### MRCoNS and Coagulase Positive Staphylococci (CoPS) Individual and Household Concomitance

In a former study Gomez-Sanz et al. (2013a,b), all coagulase positive staphylococcal (CoPS) isolates recovered from the same individuals at the same sampling (T0) were characterized (36 *S. aureus* and 18 *S. pseudintermedius*). At this stage, we aimed at making a summative and comparative analysis of the MRCoNS and CoPS concomitant carriage of individuals tested in T0 and, subsequently, of respective households. Such concomitance was also analyzed along the longitudinal study with the individuals from households with cases of IT (Supplementary File S1). Potential association of concomitant carriage, host, and/or being involved in an IT case was evaluated.

### Statistical Analysis

The characteristics of the owner and pet isolates were compared for consistent differences. Statistical analysis tests were performed in R (R Development Core Team, 2018). SCC*mec*, AMR, and *ica* locus profiles between owners and dogs were compared using the Fisher’s Exact test. Potential significant differences in MRCoNS carriage and MRCoNS/CoPS co-carriage between owners and pets at individual and household level were likewise evaluated. Correlations between presence of *ica* locus and (i) bacterial species, (ii) CC, (iii) host, and (iv) household of origin were analyzed by dependence measure of variables using multivariable Logistic Regression test. Correlations between owner and pet cohabitation and bacterial nasal carriage, as well as between involvement in **IT** cases and bacterial simultaneous carriage (MRCoNS; CoPS), at individual and household level, were likewise evaluated [variables: (i) host, (ii) presence of more than one pet per household, (iii) involvement in IT case, (iv) bacterial concomitance]. Correlation analyses were performed using the Corrplot R package. All analyses were performed at a 95% confidence interval (CIs). The degree of genetic diversity for ST and SCC*mec* types was assessed by Simpon’s Index of Diversity (SID). SID represents the probability (0 = low diversity, 1 = high diversity) that any two randomly selected species from the sample will be different. In this analysis, each ST or SCC*mec* element (*ccr*, *mec* complex combination) was considered a “type” or “species.”

## Results

### Occurrence of MRCoNS in Individuals and Households

**FIGURE 1:**
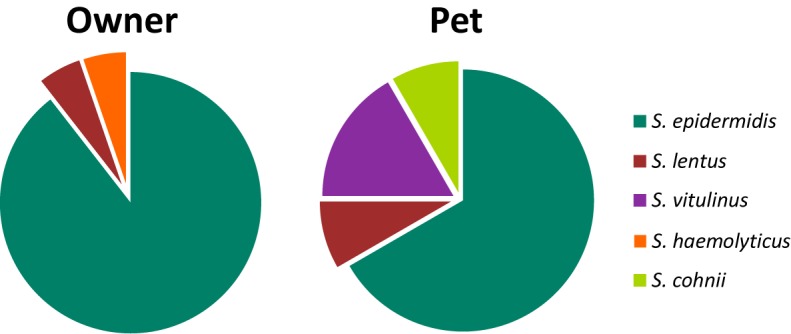
Distribution (%) of MRCoNS species recovered from the 19 positive owners (19 strains), and the 11 positive pets (12 strains). One pet (1-D1) carried one *S. lentus* and one *S. epidermidis* strain.

Thirty-one MRCoNS isolates, 19 isolates from 19 owners (27.9%) and 12 isolates from 11 pets (16.7%) (14.81% dogs, 25% cats) were detected. MRCoNS species distribution in owners and pets is shown in Figure 1. *S. epidermidis* and *Staphylococcus lentus* were detected in both owners and pets. *Staphylococcus haemolyticus* was detected in one owner only and *Staphylococcus cohnii* and *Staphylococcus vitulinus* in dogs (the latter in two cohabitant dogs). One dog (1-D1, from household no. 1) carried one *S. lentus* and one *S. epidermidis* isolate (Table 1). For both, owners and pets, *S. epidermidis* was the predominant species, accounting for 89.5 and 66.0% of strains, respectively. In total, 25% of owners and 12.1% of pets (9.3% among dogs, 25% in cats) carried MRSE.

**Table 1:** Molecular characterization of the 31 MRCoNS strains recovered from healthy owners and their pets from 20 households.

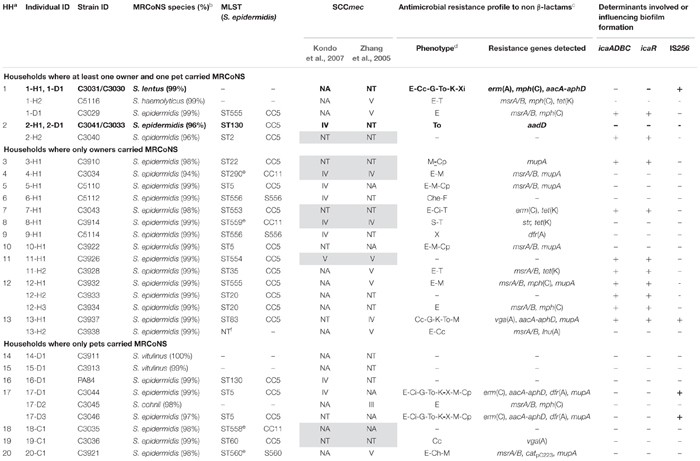

**Individuals involved in cases of Interspecies Transmission and strain characteristics are marked in bold. ^*a*^HH, household no. Households 1–2 compile data from household individuals where at least one owner and one pet carried MRCoNS Households 3–13 cluster those where only owners were positive for MRCoNS; and Households 14–20 include those where only pets carried MRCoNS. ^*b*^MRCoNS, Methicillin-Resistant Coagulase Negative Staphylococci. (%) refers to the percentage of identity to the best NCBI BLASTN hit. ^*c*^Mutations in the quinolone resistance determining regions (QRDR) of GyrA and GyrB subunits of DNA Gyrase as well as of ParC and ParE subunits of DNA topoisomerase IV are not displayed. ^*d*^E, erythromycin; Cc, constitutive clindamycin resistance; Ci, inducible clindamycin resistance; Che, clindamycin hetero resistance; T, tetracycline; G, gentamicin; To, tobramycin; K, kanamycin; S, streptomycin; M, mupirocin; X, trimethoprim/sulfamethoxazole; Xi, intermediate resistance trimethoprim/sulfamethoxazole; to Ch, chloramphenicol; Cp, ciprofloxacin; F, fusidic acid. *^*e*^Sequence type for which aroE and tpi alleles could not be amplified with standard primers.^*f*^NT, non-typeable.** Cells with gray background highlight the concordant results between both SCCmec classification schemes.*

In 20 of the 43 (46.5%) tested households there was at least one individual (either owner and/or pet) positive for MRCoNS. In two households (4.6%) (numbered 1–2) there was concurrent MRCoNS carriage of at least one owner-pet pair (Table 1). Instead, in 11 residences (25.6%) (no. 3–13) only owners were positive for MRCoNS whereas in seven households (16.3%) (no. 14–20) only pets carried MRCoNS (Table 1).

Not significant differences were observed in MRCoNS carriage among owners or pets where more than one pet was in the house (*p* = 0.7946 versus 0.4321, respectively).

### Clonal Lineages of MRCoNS Isolates

Molecular characterization of the 31 MRCoNS isolates recovered is displayed in Table 1. In total, 24 of the 25 MRSE isolates were typed by MLST, with 16 different STs detected. One human MRSE isolate (C3938) could not be typed due to reiterate lack of amplification of several of the MLST-schemed alleles (*gtr*, *pyr*, *yqil*, and *mutS*), regardless MALDI-TOF confirmed that it was *S. epidermidis*. In addition, four isolates were not typeable using the standard *aroE* and *tpi* primers^4^^,^^5^ , but did amplify with in-house designed primers (ST290 and the novel ST558, ST559, and ST560).

**FIGURE 2:**
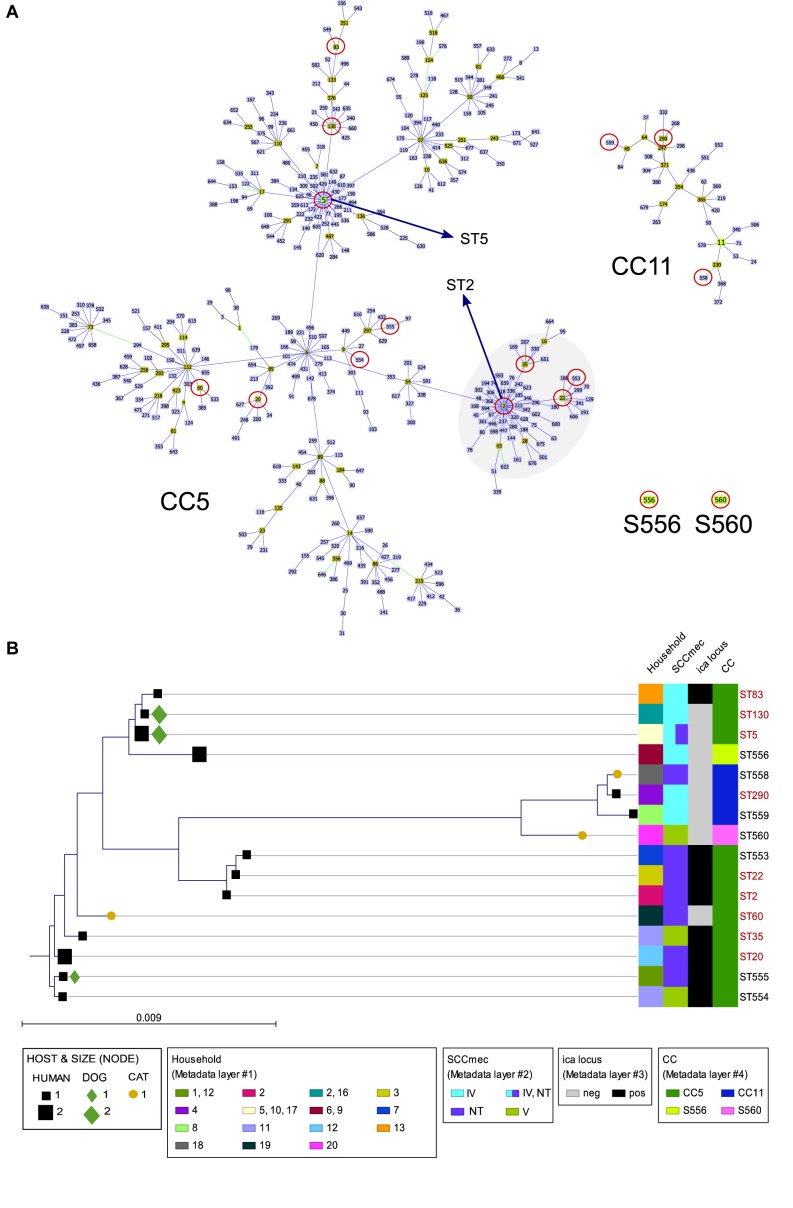
**(A)** Clustering analysis of the *S. epidermidis* STs detected in this study by goeBURST algorithm using Phyloviz 2 software (Nascimento et al., 2017). The most restricted level [level 1 – Single Locus Variant (SLV)] was used, requiring six of seven alleles shared to the linked ST. Cyan STs indicate probable ancestors (group founders) and green STs constitute subgroup founders. Blue STs correspond to STs that share the same background (CC). Circles in red indicate the STs detected in this study. Specific location of ST5 (CC5 ST primary founder) and ST2 (major subgroup founder of the cluster) within CC5 are indicated. **(B)** Distance tree of the 16 concatenate ST sequences detected among the 24 *S. epidermidis* isolates constructed using CLC Genomics Workbench 10.0.1 (https://www.qiagenbioinformatics.com/). Sequences were aligned using internal parameters, and the tree was built with a Neighbor Joining method using Jukes-Cantor as Nucleotide Distance measure, with a bootstrap analysis of 500 replicates. The bar length indicates the number of substitutions per site. STs in black color are those with new ST, either by the presence of a new allele or new allele combination.

Nine of 24 (37.5%) MRSE strains revealed novel STs (seven different ones), with either novel allele (ST553, ST554, ST555, ST556) or novel allele combination (ST558, ST559, ST560) (Supplementary Table S1). Fifteen MRSE strains (62.5%) belonged to already known STs (nine different STs). ST5 (primary ST founder of CC5) was predominant, being present in two MRSE from unrelated owners and two from related dogs. Most MRSE belonged to CC5 (75%), which is the major group within the *S. epidermidis* MLST scheme, three strains belonged to CC11 (12.5%) and three strains were singletons [S556 (8.3%) and S560 (4.2%)] (Figure 2A). All previously known STs (ST2, ST35, ST22, ST60, ST20, ST130, ST83, and ST290) represented subgroup founders (by default settings, i.e., an ST with at least three links to other STs, including the link to its assumed progenitor), with ST2 as the biggest subgroup founder within CC5 (formerly compiling CC2) (Figure 2A).

The distance tree of the 16 concatenate ST sequences detected among the 24 MRSE strains revealed high profile diversity. All cases were concordant with the CC and STs subgroup clusters (based on allelic profile) represented by Phyloviz2 clustering using the goeBURST algorithm, except for ST35, which formed an independent branch from the closest variants (ST2, ST22, and ST553). Remarkably, all four STs that could not be amplified using the standard primers (all 3 CC11 and S560) clustered together in a distant branch from the rest of STs (Figure 2B).

All canine MRSE strains exhibiting STs were also detected in owners (1 ST155, 2 ST5, 2 ST130, all CC5), while all feline (ST60-CC5, ST560-S560, ST558-CC11) and some human MRSE strains were unique (Figure 2B).

The Simpon’s Index of Diversity (SID) was remarkably high (0.96), reflecting a 95.6% chance of randomly picking two strains from the sample cohort that are different.

### *ccr* and *mec* Complex Diversity Among MRCoNS Isolates (SCC*mec* Profile)

Based on a scheme by Kondo et al. (2007), high diversity of *ccr* types, *mec* complexes and *ccr-mec* complex combinations were detected among the 31 studied isolates (Table 1). *ccrAB2* (*n* = 21) and *mec* complex A (*n* = 13) were predominant within their respective category (see Supplementary Table S2). All eight SCC*mec* cassettes carrying *ccrC* presented additional *ccr* genes (*ccrAB2*, *n* = 5; *ccrAB1*, *n* = 2; or *ccrAB1*+*ccrAB2*, *n* = 1) (Supplementary Table S2). More than one *ccr* type was detected in 11 isolates (35.5%). A total of 21 SCC*mec* cassettes were either NT or NA (67.7%), nine were SCC*mec* IV (29%), and one was SCC*mec* V (3.2%).

According to scheme by Zhang et al. (2005), 20 strains were either SCC*mec* NT or NA (64.5%), seven were SCC*mec* V (22.6%), three were SCC*mec* IV (9.7%), and one was SCC*mec* III (3.2%). Four strains were positive for more than one SCC*mec* cassette.

Eight of 31 strains (25.8%) were concordantly typed with both typing schemes (Table 1 and Supplementary Table S2). Among them, SCC*mec* NT was predominant (*n* = 4), followed by SCC*mec* IV (*n* = 2) and SCC*mec* V and SCC*mec* NA (one each), respectively. Both schemes categorized seven additional cassettes in different strains, with a SID of 0.89 by Kondo et al. (2007) and SID 0.71 by Zhang et al. (2005). In total, as a consensus of both schemes, 18 strains of SCC*mec* were NT (58.1%), 10 NA (32.3%), two SCC*mec* IV (6.5%), and one SCC*mec* V (3.2%) (Supplementary Table S2).

Comparing owner versus pet MRCoNS isolates by Kondo et al. (2007), SCC*mec* NT or NA were predominant among both human and animal strains (combined 63.2% for owners versus 91.7% for pets) (*p* = 0.02203). SCC*mec* IV was the most commonly known SCC*mec* cassette among both host isolates (six from humans, 31.6%; three from dogs, 25%), while SCC*mec* V was only detected in two owners (10.5%).

According to both schemes performed, one MRSH from an owner and one MRSE from her pet (1-H2 and 1-D1, household 1) shared the same SCC*mec* cassette (Table 1 and Supplementary Table S2).

### Antimicrobial Resistance (AMR) Pattern

**FIGURE 3:**
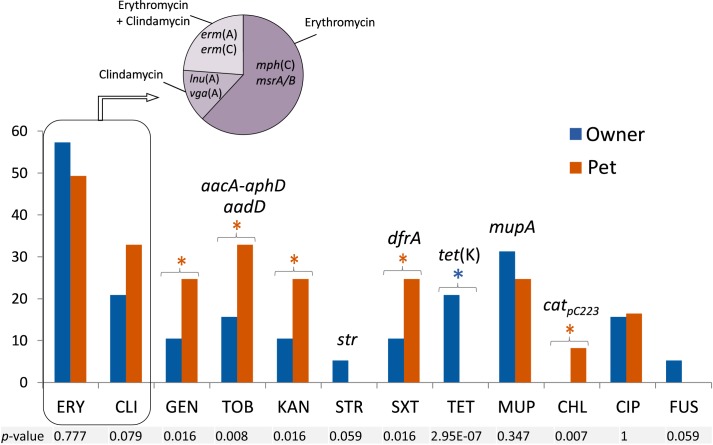
Percentage of resistance to non β-lactams and antimicrobial resistance genes detected among the 31 MRCoNS isolates investigated in T0. FUS, fusidic acid; CHL, chloramphenicol; TET, tetracycline; CIP, ciprofloxacin; SXT, co-trimoxazole; GEN/TOB/KAN/STR, gentamicin/tobramycin/kanamycin/streptomycin; MUP, mupirocin; ERY/CLI, erythromycin/clindamycin. All isolates were susceptible to vancomycin and linezolid. Individual *P*-value (Fisher’s Exact Test for count data) to account for significant difference at 95% confidence interval is indicated at the bottom of the histogram. Asterisks (blue or red) above the bars represent those agents for which statistical differences were detected, with the asterisk color remaking the host (owner or pet, respectively) of the bacteria involved in the significance.

Prevalence of resistance to non β-lactams among human and animal isolates, as well as the detected resistance genes, is shown in Figure 3. Erythromycin resistance [*erm*(A), *erm*(C)] was the most common pattern (51.6% of isolates), followed by mupirocin (*mupA*) (29%) and clindamycin [*vga*(A), *lnu*(A)] (29%) resistance. Subsequently, MLS was the antimicrobial class to which most strains exhibited resistance. Mupirocin resistance was only present in MRSE strains (36% of MRSE). Inducible clindamycin resistance was only observed in the three isolates carrying the *erm*(C) gene (see Table 1).

Mutations identified in the QRDR of the *gyrA*, *parC* and *parE* genes of the five MRSE ciprofloxacin resistant strains are summarized in Table 2. All detected substitutions are displayed in Supplementary Table S3. No mutation was observed in any strain within the *gyrB* gene sequence region. The most common mutation was Ser84Phe (5/5) and Ser84Tyr (3/5) in GyrA and ParC, respectively.

**Table 2:** Mutations in the quinolone resistance determining regions (QRDR) of GyrA (DNA Gyrase), ParC, and ParE (DNA topoisomerase IV) of the quinolone resistant strains.

Strain	GyrA		ParC		ParE	
			
	Synonymous substitution	Non-synonymous substitution	Synonymous substitution	Non-synonymous substitution	Synonymous substitution	Non-synonymous substitution
C3044, C3046	**S84F**	P34P, V74V	**S80F, D84G**	V70V, Q73Q, G104G	–	L411L, L430L, L442L, V458V
C3910	**S84F, E88K**	P34P, V74V	**S80Y, D84Y**	V70V, Q73Q, G104G	–	L411L, L430L, L442L, V458V
C3922	**S84F, E88K**	P34P, V74V	**S80Y, D84Y**	V70V, Q73Q, G104G	–	L411L, L430L, L442L, V458V
C5110	**S84F**	P34P, V74V	**S80Y**	V70V, Q73Q, G104G	–	L411L, L430L, L442L, V458V


*GyrB is not represented given that no mutations were observed. Synonymous substitutions are marked in bold.*

Resistance to aminoglycosides (*p* = 0.008–0.016), co-trimoxazole (*p* = 0.016) and chloramphenicol (*p* = 0.007) was significantly higher in animal isolates (with the latter being exclusively detected in pets), whereas resistance to tetracycline was only present and abundant in owner isolates (*p* = 2.95E-07). Resistance to fusidic acid and streptomycin were only detected in human isolates at low rates, but no significant differences were observed with the Fisher’s Exact test.

Remarkably, one methicillin-resistant *S. lentus* (MRSL) clone (isolates C3030 and C3031, from owner 1-H1 and cohabitant dog 1-D1) showed intermediate resistance to trimethoprim and co-trimoxazole but did not harbor any of the trimethoprim resistance genes so far described in staphylococci. The human MRSE-S556 strain (C5112) also showed hetero-resistance to clindamycin but was negative for the corresponding genes tested. This strain was also resistant to fusidic acid and lacked the acquired *fusB* and *fusC* genes.

Significant differences were observed between the rate of owners and pets carrying MDR MRCoNS isolates (68% versus 33%) (*p* = 1.205E-06). In total, 54.84% of isolates were MDR.

### Presence of Determinants for Biofilm Formation

A total of 32.3% of isolates were positive for the genes enclosed within the *ica* locus (*icaADBC*) as well as for the *icaADBC* transcriptional regulator *icaR* (Table 1); all of which were MRSE of the CC5 lineage (see Figure 2B). If divided by the bacterial host, 47.4% of human isolates and a single MRSE canine strain (C3029) (8.3%) were positive (*p* = 4.49e-10). Subsequently, the presence of the *ica* locus gene cluster in human MRSA-CC5 isolates was strongly positively correlated. Through logistic regression analysis, positive association was observed between presence of the *ica* locus and owners, only when the variable household of origin was not considered in the equation (association was observed at 0.1 significance code otherwise).

The IS*256* was detected in four *icaADBC*-negative isolates (12.9%). These isolates also contained the bifunctional aminoglycoside resistance *aacA-aphD* gene, which is normally enclosed within Tn*4001* (IS*256*_*aacA-aphD*_IS*256*).

### Owner/Pet MRCoNS IT Cases and Longitudinal Overview

**FIGURE 4:**
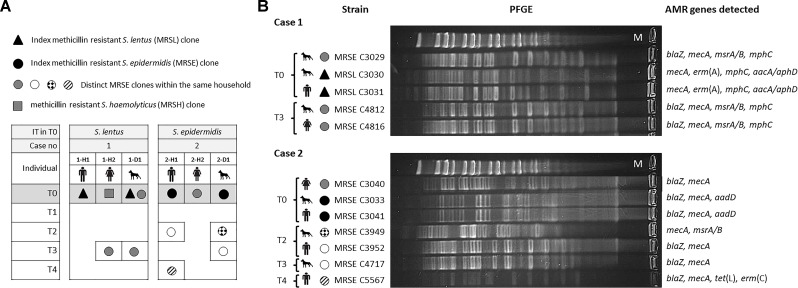
**(A)** Schematic representation of the methicillin resistant coagulase negative staphylococcal carriage dynamics of both households investigated along 1 year. IT, bacterial species responsible for interspecies transmission. T0–T4 indicate the different sampling times along the sampling year. Individuals are named H (for human) or D (dog) followed by the case number (1 or 2) and a lower-case letter to differentiate subjects per household. **(B)** Pulsed-field Gel Electrophoresis (PFGE) profile of genomic DNA digested with SmaI restriction enzyme of isolates recovered from individuals involved in both cases of possible direct interspecies transmission. Upper lane in PFGE per case corresponds to MidRange PFGE Marker (New England Biolabs). Antimicrobial resistance (AMR) genes detected in each strain are also indicated.

Based on all molecular techniques performed, two cases of IT were identified in the owner and cohabitant dog in two unrelated households (4.7% of tested residences; 10% of households with MRCoNS-carrying individuals): (i) a MDR MRSL clone (1-H1 and 1-D1), resistant to erythromycin/clindamycin and gentamicin/tobramycin/kanamycin; and (ii) a MRSE-ST130-CC5 clone (2-H1 and 2-D1) resistant to tobramycin/kanamycin (Table 1 and Figure 4).

According to the 1 year longitudinal study, in case 1, sporadic carriage by the involved MRSL clone was observed in both individuals (1-H1, 1-D1). Instead, the involved dog (1-D1) also carried a MRSE ST155-CC5-SCC*mec*V strain (resistant to erythromycin) in T0 which was also present when sampling T3 in the same animal (intermittent carrier of such clone) as well as in the other cohabitant owner (1-H2), representing an additional *S. epidermidis* sporadic IT case (Figure 4). In total, three different MRCoNS species (*S. lentus, S. epidermidis, S. haemolyticus*) and one clone of each were detected along the sampling year. Dog 1-D1 carried two of these clones while the owners carried one clone each.

In case 2, sporadic carriage by the involved MRSE ST130-CC5 clone was also observed. Notably, the same owner and dog (2-H1, 2-D1) carried an identical non-concurrent MRSE clone (only resistant to β-lactams) in different samplings: T2 for the owner and T3 for the dog (Figure 4), indicating transient carriage and suggesting that such a clone might be circulating within the household. Along the sampling year, these two subjects revealed to be intermittent carriers of different *S. epidermidis* clones with different resistance patterns (Figure 4). In total, a single MRCoNS species (*S. epidermidis*) was detected throughout the sampling year, however, five different MRSE clones were observed, three of them found in dog 2-D1, three in owner 2-H1 and a single clone in owner 2-H2.

None of the individuals, from both cases, were persistent carriers by any of the recovered MRCoNS strains. None of the IT-involved isolates in T0 exhibited any of the genes of the *ica* locus. However, the MRSE C3029 clone (from case 1), which carried the *ica*-locus, was detected again in this animal and one owner in T3 (IT case).

The dynamics of all CoPS staphylococci detected in the same samplings (T0–T4) are described in the Supplementary File S1 as well as in Supplementary Figure S1.

### Individual and Household MRCoNS and/or CoPS Concomitance

Eighty-five staphylococcal strains [MRCoNS (*n* = 31) and CoPS (*n* = 54)] (Gomez-Sanz et al., 2013b) from the 68 positive individuals recovered at the same sampling point were compared here (Supplementary Table S4). This comprehensive picture revealed a total of nine cases of IT (two MRCoNS, 7 CoPS) at sampling T0 (11.9% of subjects coming from 18.6% of tested households) (Gomez-Sanz et al., 2013a,b). Altogether, 55.9% of owners and 45.5% of pets were positive for MRCoNS and/or CoPS (Supplementary Table S5).

**FIGURE 5:**
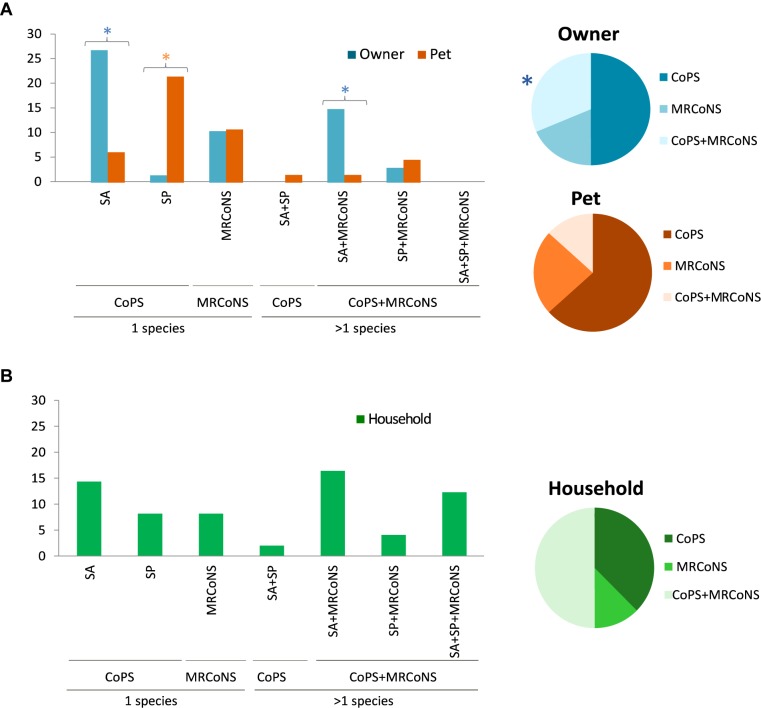
**(A)** Left panel, bar chart showing the percentage of owners and pets that carried Coagulase Positive Staphylococci (CoPS), i.e., *S. aureus* (SA) and/or *S. pseudintermedius* (SP); MRCoNS; or CoPS + MRCoNS in sampling T0 (Gomez-Sanz et al., 2013b). Right panel, graphical view of the distribution of CoPS and/or MRCoNS detected among the individuals positive for such bacterial species. **(B)** Left panel, bar chart displaying the percentage of households with individuals positive for CoPS (SA, SP), MRCoNS or CoPS + MRCoNS in sampling T0. Right panel, graphical representation of the distribution of CoPS and/or MRCoNS detected among the households with individuals positive for such bacterial species. Colored stars indicate values with significant differences between human and animal strains.

Single presence of CoPS was the most common pattern, with owners and pets predominantly carrying only *S. aureus* (26.5%) or *S. pseudintermedius* (22.7%), respectively (Figure 5A). The carriage rate of MRCoNS as the single species recovered was similar in owners and pets tested (ca. 10.5%) (Figure 5A). Alternatively, 17.7% of owners and 6.1% of pets simultaneous carried both bacterial types (*p* = 0.015) (Table 3). Concomitant carriage of MRCoNS and *S. aureus* was significantly higher among owners than pets (14.7% versus 1.5%), while no significant differences were detected for co-carriage of MRCoNS and *S. pseudintermedius* (2.9% versus 4.6%) (Figure 5A and Supplementary Table S5).

**Table 3:** Individuals concomitantly carrying at least one MRCoNS and one CoPS isolate ranked by household type (based on carriage and IT) and major strain characteristics.

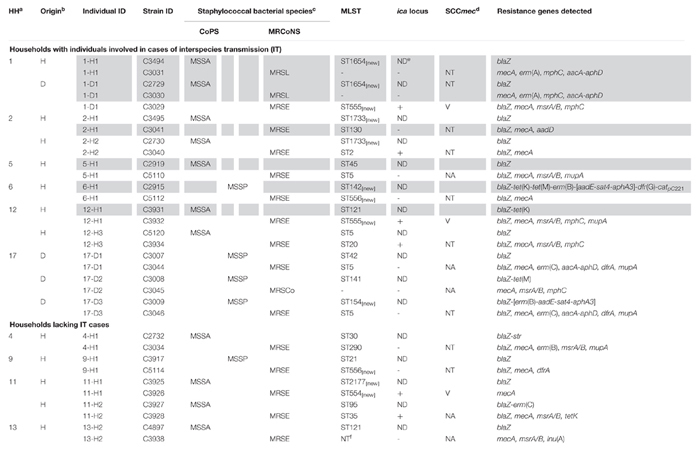

**Cells highlighted in faint gray indicate individuals directly involved in cases of interspecies transmission by any of tested staphylococcal type (from this study and Gomez-Sanz et al., 2013b). ^*a*^HH, household no. Nomenclature based on the current study (different nomenclature was used in Gomez-Sanz et al., 2013a,b). Households 1, 2, 5, 6, 12, and 17 include individuals involved in cases of Interspecies Transmission (including by CoPS, Gomez-Sanz et al., 2013b). Households 4, 9, 11, and 13 group individuals where only owners co-carried MRCoNS and CoPS. ^*b*^H, Human; D, dog. ^*c*^CoPS, coagulase positive staphylococci; MSSA, methicillin-susceptible S. aureus, MSSP, methicillin-susceptible S. pseudintermedius; MRSP, methicillin-resistant S. pseudintermedius; MRCoNS, methicillin-resistant coagulase negative staphylococci; MRSL, methicillin-resistant Staphylococcus lentus; MRSE, methicillin-resistant Staphylococcus epidermidis; MRSCo, methicillin-resistant Staphylococcus cohnii. ^*d*^Consensus SCCmec cassette. ^*e*^ND, non-determined. ^*f*^NT, non-typeable.**

Eleven of the 17 MRCoNS (64.7%) strains involved in the simultaneous carriage were MDR and six of 17 (35.3%) contained the *ica*-locus genes, involved in biofilm formation (Table 3). IT cases were more common among individuals with concomitant carriage (6/16, 37.5%) (*p* = 0.004).

At the household level, based on the strains recovered from individuals tested, 32 households were positive for any of the tested bacterial species (74.4%; 34.9% positive for one bacterial type, 39.5% positive for both MRCoNS and CoPS) (Figure 5B). Co-presence of *S. aureus* and MRCoNS was the most predominant pattern (18.6%), followed by *S. aureus* alone (16.3%), and co-presence of *S. aureus*, *S. pseudintermedius* and MRCoNS (14%). Considering the 32 positive residences, *S. aureus* was the predominant species among households with a single bacterial type (21.9%), and half (50%) presented both MRCoNS and CoPS bacterial types (Figure 5B).

In total, 23.3% of households contained individuals simultaneously harboring both bacterial types (Table 3). Half (5/10) of these households enclosed subjects directly involved in IT cases (*p* = 0.011). Further, all four pets and seven of the 12 owners who tested positive for concomitant MRCoNS and CoPS (11/16, 68.8%) originated from households where IT cases occurred, even if they were not the individuals directly involved in the case (Table 3).

### Association Between MRCoNS and CoPS Concomitance, IT Cases, and Host

Logistic regression analysis confirmed a strong positive correlation between individual staphylococcal concomitance and involvement in IT case (0.001 significance code). A positive association (0.05 significance code) was observed between concomitance and owners, only when the household of origin was not considered in the equation (association was observed at 0.1 significance code otherwise).

No significant differences were observed between the presence of more than one animal in the house (animal cohabitation) and (i) staphylococcal carriage (*p* = 0.3145 for pets, *p* = 0.1644 for owners), or (ii) MRCoNS and CoPS individual co-carriage (*p* = 1 for pets, *p* = 0.7781 for owners).

## Discussion

The present study provides novel information on frequency, population structure, genetic diversity, AMR and virulence potential among MRCoNS from companion animals and their owners within the household, as well as on staphylococcal human-pet interaction and persistence. The MRCoNS carriage rate detected among healthy owners (28%) is remarkably higher than those detected in former studies among healthy individuals in non-healthcare settings, with rates ranging between 7 and 17% (Barbier et al., 2010; Rolo et al., 2012; Du et al., 2013; Abadi et al., 2015; Xu et al., 2018). Higher nasal MRCoNS rates (30, 47–51%) were detected in Japanese children in day-care centers and kindergartens (Jamaluddin et al., 2008) and among a remote population in French Guiana (Lebeaux et al., 2012). On the other hand, a recent international study on nasal staphylococcal colonization among healthcare workers from 75 different countries revealed a nasal MRCoNS carriage rate of 21.4% (Morgenstern et al., 2016). All these data reflect that nasal distribution of MRCoNS markedly depend on the cohort studied. Remarkably, scarce data are available on the nasal MRCoNS colonization rate among pet owners, and or the animal-owner contact as a possible contributor in increased MRCoNS carriage. Only a couple of recent studies analyzed the risk factors of MRS carriage among individuals in contact with companion animals (Han et al., 2016; Rodrigues et al., 2018). Rodrigues et al. (2018) reported an overall prevalence of MRCoNS of 54.2% among healthy humans in professional daily contact with companion animals in Portugal. In this report, being a veterinary professional was identified as a risk factor for methicillin-resistant staphylococcal carriage (both CoNS and CoPS) colonization (Rodrigues et al., 2018). The relatively high MRCoNS rate detected here might therefore be due, at least partially, to direct pet-human contact, and might be considered as a risk factor for colonization. However, the lack of a “control” population in the current study forces us to interpret these data with caution. Among pets, very sparse data are available on the specific nasal MRCoNS rates. Lower rates (1–15%) than those detected here (17%) have been observed among healthy dogs from several body sites (nasal, rectal, oral, anal, belly) (Vengust et al., 2006; Bagcigil et al., 2007; Aslantas et al., 2013; Gandolfi-Decristophoris et al., 2013; Garbacz et al., 2013; Chah et al., 2014; Davis et al., 2014; Wedley et al., 2014; Siugzdaite and Gabinaitiene, 2017). Interestingly, MRCoNS was isolated from 42% of healthy non-vet visiting and non-antimicrobial treated Labrador retrievers in the United Kingdom (Schmidt et al., 2014). In the latter study, both nasal and perineal samples were collected, suggesting that different sampling methodologies may affect observed prevalence.

In humans, *S. epidermidis* is the most frequently recovered staphylococcal species, colonizing the body surface (Becker et al., 2014; Schmidt et al., 2014). Moreover, the *S. epidermidis* group (predominantly *S. epidermidis* and *S. haemolyticus*) is the most significant species within CoNS representing one of the major nosocomial pathogens (Becker et al., 2014). As such, MRSE was the MRCoNS predominant species detected (25%, 99% of human strains). *S. epidermidis* was also the predominant species among tested animals, with an overall prevalence of 12.1% (9.3% among dogs versus 25% in cats), corresponding to 66% of strains. A diverse range of MRCoNS species have been detected among dogs, such as *Staphylococcus sciuri*, *Staphylococcus warneri, S. lentus*, *S. vitulinus*, or *Staphylococcus fleurettii* (Bagcigil et al., 2007; Becker et al., 2014; Chah et al., 2014; Davis et al., 2014; Schmidt et al., 2014; Siugzdaite and Gabinaitiene, 2017). Regardless *S. epidermidis* has a more defined role in humans; it may also form part of the normal microbiota of animals and, although at lower rates, has been detected as the predominant CoNS species among healthy dogs (Aslantas et al., 2013; Schmidt et al., 2014; Han et al., 2016). Nevertheless, *S. epidermidis* is a predominantly human associated bacterium and the observed distribution here may be influenced by the human-pet direct or indirect contact within the household.

*S. epidermidis* is the most studied species within CoNS and it is characterized by pronounced genomic diversity (Becker et al., 2014). This agrees with the high diversity of MRSE STs detected (SID of 0.96). In spite of the scattered data available on MRSE lineages from healthy individuals, former reports have also reported high rates of novel STs among *S. epidermidis* isolates (Xu et al., 2018), evidencing high intra-species diversity. MRSE CC5 was predominant, clustering 75% of MRSE isolates from owners. This clonal lineage (with ST5 as primary founder) represents the biggest group within the MLST scheme for this species. MRSE ST2 and ST22, among others, currently enclosed within CC5 but traditionally constituting its own CC (CC2), have been shown to be predominant among hospital environments (Miragaia et al., 2007; Rolo et al., 2012; Cherifi et al., 2013; Becker et al., 2014; Widerstrom et al., 2016; Gordeev et al., 2017). In the community, a high diversity of STs have been identified among healthy individuals (Miragaia et al., 2007; Rolo et al., 2012; Cherifi et al., 2013; Becker et al., 2014; Widerstrom et al., 2016; Gordeev et al., 2017). In contrast, recent studies have revealed a high diversity of lineages among MRSE from both clinical and healthy individuals, with either no increased abundance of CC5 strains among clinical isolates (Jena et al., 2017) or with CC5 predominance in both settings (Rolo et al., 2012; Du et al., 2013). This may be due to the fact that most STs already cluster into CC5 by eBURST/goeBURST analyses, which may hamper attempts to identify lineages that might be associated with different regimes (Thomas et al., 2014). For this reason, a couple of recent studies implemented a Bayesian clustering approach to appraise the real species-wide population structure and ecology of *S. epidermidis*, detecting six genetic cluster (GCs) based on their adaptation to nosocomial or commensal lifestyles (Thomas et al., 2014; Tolo et al., 2016). Following this classification for the already known STs, (i) ST2 and ST22 were more suited to a nosocomial lifestyle (GC5); (ii) ST290 to a more commensal lifestyle (GC4), (iii) ST5, ST83, and ST130 were adapted to a more generalist-to-non-hospital sources (GC1); and (iv) ST20, ST35, and ST60 were better suited for generalist-to-infection-associated lifestyles (GC6).

Very scarce data are available on MRSE lineages among pets. A few studies among clinical samples detected ST5 and/or ST2 (both CC5) as predominant, in line with data from humans (Kern and Perreten, 2013; Weiss et al., 2013; Couto et al., 2016). However, data on the circulating MRSE lineages in the community and whether they reflect the human circulating lineages within a target system, are lacking. Here, clear clustering of human and canine strains was observed, as all STs detected among dogs were also detected among different owners from different households. This lack of host tropism of specific lineages suggests the adaptability potential of MRSE to different hosts within a shared habitat and/or the easiness of host sporadic acquisition of circulating lineages. In contrast, the three STs detected among MRSE from feline isolates were unique. This might indicate that, while dogs tend to share the same clonal lineages as owners, cats might pose feline-associated lineages. Further studies with a bigger sample size are needed on the ecology of MRCoNS and MRSE among different inhabitant species, and how cohabitation may influence host staphylococcal profiles.

High diversity of SCC*mec* types was detected, most being either NT or NA (90.3%, 28/31). These values are notably higher than those detected among both clinical and community MRCoNS human isolates (Barbier et al., 2010; Lebeaux et al., 2012; Aslantas et al., 2013; Abadi et al., 2015; McManus et al., 2015). This high rate may be partially due to the higher discriminatory power of using two schemes. Remarkably, slightly similar values (83%) were recently detected among MRSE from the nares of neonates at hospital admission (Salgueiro et al., 2017). It is challenging to define whether the NTs cassettes identified here are identical to those previously described as NTs, due to variances in typing methods and the lack of full analysis of the genetic organization and composition of these elements. For this, further in-depth analyses, such as whole genome sequencing (WGS), are definitively needed.

Lack of robust concordance was observed between results obtained by both schemes, with guidelines from Kondo et al. (2007) showing a remarkable high diversity index (SID 0.89 versus 0.71), and reflecting the high intergenic diversity within MRCoNS cassettes. SCC*mec* IV was the predominant typeable cassette for both owner and pets, and despite, additional cassettes have been sporadically detected, it is also the most prevalent cassette among humans and companion animals (Ruppe et al., 2009; Barbier et al., 2010; Lebeaux et al., 2012; Aslantas et al., 2013; Kern and Perreten, 2013; Park et al., 2013; Weiss et al., 2013; Becker et al., 2014; Abadi et al., 2015; McManus et al., 2015; Couto et al., 2016).

Several *ccr* genes were detected in 35.5% of strains, showing a high variety of site-specific recombinases among MRCoNS. The possibility that primers are not specific enough for potential new *ccr* cannot be discarded. Further, *ccr2* and *ccrC* were co-present in all but one detected cases, suggesting that clustering of both *ccr* genes might imply and adaptive advantage. Further analyses should be performed to unveil the real presence and functionality of redundant *ccr* genes, and whether this implies an adaptive advantage under specific conditions. The high SCC*mec* variability, lack of typeability and presence of novel *ccr* and *mec* complex combinations reflect an ever-increasing complexity among SCC*mec* cassettes among CoNS from healthy individuals. Such mobile elements may represent a source for the potential transfer to concurrent staphylococci sharing the same niche. In this study, however, transmission of β-lactams resistance between MRCoNS and CoPS appears negligible among the population tested.

Macrolides-Lincosamides-Streptogramins (MLS), especially erythromycin, was the antimicrobial class for which most strains exhibited resistance among owners and pets (64.5%). MLS are important antibiotics for treatment of staphylococcal infections in both humans and animals (Guardabassi et al., 2004; Bagcigil et al., 2007). Subsequently, it is not surprising that MLS resistance is common among staphylococci in the community (Aslantas et al., 2013; Gandolfi-Decristophoris et al., 2013; Garbacz et al., 2013; Wedley et al., 2014; Couto et al., 2016; Han et al., 2016). Of note, combined resistance to erythromycin and clindamycin is the most common MLS pattern among CoPS isolates (Gomez-Sanz et al., 2013a,b), however, most MRCoNS isolates here were either resistant only to erythromycin or to clindamycin. This pattern reflects the potential differential ability to acquire different resistance genes between CoPS and MRCoNS populations.

Resistance to Aminoglycosides, co-trimoxazole and chloramphenicol was significantly higher among pet isolates. Resistance to these agents, especially to aminoglycosides and trimethoprim, has been reported as common among staphylococci of healthy dogs, and these agents are used extensively in hospital and veterinary settings (Guardabassi et al., 2004; Penna et al., 2010; Chah et al., 2014; Wedley et al., 2014; McManus et al., 2015; Han et al., 2016; Conner et al., 2018). Interestingly, Tetracycline was only detected among human strains, while this antibiotic is widely used in both human and animal medicine (Guardabassi et al., 2004). The lack of resistance among animal strains differs from former studies among both healthy and clinical canine isolates, with rates ranging between 40 and 81% (Aslantas et al., 2013; Kern and Perreten, 2013; Chah et al., 2014; Wedley et al., 2014; Couto et al., 2016; Siugzdaite and Gabinaitiene, 2017). Such differences are most likely due to the groups studied and the geographical area of the sampling. Further research is therefore needed to ponder these profiles as common trends among MRCoNS from healthy pets in Spain.

Interestingly, mupirocin and ciprofloxacin resistance were associated to MRSE and only detected in this species (36 and 20%, respectively). This association is relevant and may reflect a higher exposure of MRSE strains to these agents, which might be partially due to the higher pathogenic potential of this CoNS species. Little is known about the real prevalence of mupirocin resistance (MR) among the CoNS population (Becker et al., 2014), and even less among staphylococci from pets. A couple of studies have detected lower resistance levels, even among clinical samples (8-20%) (Aslantas et al., 2013; Kern and Perreten, 2013; Wedley et al., 2014; Couto et al., 2016). The high rate of mupirocin resistance detected among MRSE (both in owners and pets) is alarming as it is the key antibiotic used for decolonization of methicillin-resistant *S. aureus* (Becker et al., 2014).

MDR was high (54.8%) and significantly higher among human isolates (68.4% versus 33.3%). This difference may again reflect higher exposure of humans to antimicrobial therapy or the clinical settings, or to the coexistence of resistance strains within the same ecological niche, which may favor the horizontal transfer of their mobile elements. Diverse MDR values have been observed among staphylococci from healthy dog owners and pets (17–93%), with most studies reporting very high MDR values (Gandolfi-Decristophoris et al., 2013; Garbacz et al., 2013; Wedley et al., 2014; Han et al., 2016; Siugzdaite and Gabinaitiene, 2017; Conner et al., 2018). Therefore, MRCoNS from healthy owners and pets represent a reservoir for AMR gene transfer in the community and may hamper successful treatment of staphylococcal infections in both animals and humans.

A relatively high rate of isolates (32%) was positive for *ica* locus, which is one of the key elements involved in the early stages of biofilm formation (intercellular adherence and cell agglutination) (Becker et al., 2014). Several studies have shown that *S. epidermidis* from healthy individuals or community environments less frequently carry *ica*ADBC-cluster genes, in comparison to clinical samples or hospital-associated environments (Fey and Olson, 2010; Becker et al., 2014; Szczuka et al., 2016; Seng et al., 2017). The rates detected here are therefore outstanding and reflect that MRCoNS strains spread in the community pose notable virulence properties. Interestingly, in the current study, *ica*ADBC was positively correlated with human MRSE CC5 isolates (47.7%). Harris et al. (2016) recently identified *S. epidermidis* of this lineage as *ica*ADBC-containing biofilm producers. However, they could not establish lineage-biofilm formation associations, as the genes involved were present in divergent lineages, showing evidence for horizontal gene transfer. Alternatively, although most cases of biofilm-forming CoNS isolates and biofilm-associated infections containing the *ica*-locus are from *S. epidermidis*, other CoNS species have occasionally been detected to form biofilms and to contain this operon (Szczuka et al., 2016; Seng et al., 2017).

To the best of our knowledge, this is the first study addressing the occurrence and persistence of MRCoNS transmission between owners and their pets. Two cases of IT were detected in T0 (4.7%). Diverse sequential MRCoNS clones were observed on the longitudinal approach among tested individuals, revealing a MRCoNS existent flow in the household setting and the vector-role of dogs for human staphylococcal acquisition, and vice verse. In addition, dog 1-D1, involved in the MRSL IT case in T0, was also positive for a MRSE *ica*-locus positive strain (C3029 MRSE-ST155-CC5-SCC*mec*V), which was responsible for an additional case of IT in T3 (9 months after first sampling). *S. epidermidis* is a human related species, whereas *S. lentus* is considered animal-associated (Becker et al., 2014). Subsequently, the MRSE-involved IT cases here are suggested to have an anthropozoonotic origin, whereas the MRSL case may be regarded as of zoonotic origin. These data provide evidence that MDR and virulent MRCoNS strains can be exchanged and at least temporarily persist between owners and in-contact pets, contributing to the dissemination of resistant staphylococci, with the subsequent risk of infection. To this end, the household environment could also play a role as source for MRCoNS and persistence in the sampled population, as recently reported from community environments (20.5%) (Seng et al., 2017).

To our knowledge, this is also the first study addressing simultaneous nasal carriage of CoPS and MRCoNS in owners and their pets. A single study, focused on the occurrence of CoPS and MRCoNS in dogs, observed slightly higher carriage rates to the ones detected here (45.5%), with 55% of animals positive for CoPS and/or MRCoNS (Wedley et al., 2014). However, CoPS and MRCoNS co-carriage was as low as 2.2%, in comparison to the 6.1 and 16.2% detected among our animal and human population, respectively. Alternatively, although owners and pets differed in the CoPS predominant species when occurring alone or in concomitance with MRCoNS, no significant differences were observed when addressing the single presence of MRCoNS. Again, this might indicate a less prone host-tropism among MRCoNS than among *S. aureus* or *S. pseudintermedius*, or the capacity to adapt or temporarily coexists in different hosts. In addition, owners tend to simultaneously carry both bacterial types. Based on the strong association between involvement in an IT case and CoPS-MRCoNS simultaneous carriage, we reveal that owner-pet inhabitance favors the coincident coexistence of the staphylococcal species with high virulence potential and/or MDR pattern. This scenario does not only disclose an exchange of relevant bacteria between owners and pets, but also paves the way for the exchange of AMR and virulence factors between concomitant strains. Whether these owner-pet exchanged microbes have a true niche on these pairs, versus transient detection after direct or indirect contact, is unknown. However, these results suggest that owner-pet inhabitance may significantly shape the staphylococcal population composition of one another.

## Conclusion

MRCoNS, especially MRSE, are common colonizers of healthy owners and pets. They show high clonal diversity, represent a reservoir of AMR genes and pose IT potential. The detection of MRSE clonal lineages that circulate in human hospitals and the community suggests that companion animals can contribute to the dissemination of highly successful human clones. Due to the sequential MRCoNS clones detected in owners and pets over time, more longitudinal studies are required to distinguish between persistent colonization, transient carriage or mere contamination, as well the implication of what the different statuses can imply for public health. Individuals involved in cases of IT revealed to be prone to simultaneous CoPS-MRCoNS co-carriage. These data highlight the importance of companion animals as reservoirs of important MDR opportunistic pathogens, which can be transferred to in-contact individuals. Further epidemiological studies including samples from environmental sites are needed to elucidate the conditions by which MRCoNS are propagated within household settings, as well as to confirm owner and pet cohabitation as a risk factor for the acquisition and subsequent infection by MDR staphylococci.

## Data Availability

All datasets generated for this study are included in the manuscript and/or the Supplementary Files.

## Author Contributions

EG-S, CT, and MZ conceived and designed the experiments. EG-S, SC, and LR-R performed the experiments. EG-S analyzed the data and wrote the manuscript. All authors contributed to manuscript revision, read and approved the submitted version.

## Conflict of Interest Statement

The authors declare that the research was conducted in the absence of any commercial or financial relationships that could be construed as a potential conflict of interest.

## Footnotes

1 https://pubmlst.org/sepidermidis/

2 http://goeBURST.phyloviz.net

3 http://www.sfm-microbiologie.org/

4 https://pubmlst.org/sepidermidis/

5 http://sepidermidis.mlst.net
