# Supplementary material for: Clonally Diverse Methicillin and Multidrug Resistant Coagulase Negative Staphylococci Are Ubiquitous and Pose Transfer Ability Between Pets and Their Owners
Source: Front Microbiol. 2019 Mar 26;10:485. doi: 10.3389/fmicb.2019.00485 (PMC6443710; doi:10.3389/fmicb.2019.00485)
Supplement: Supplementary file 1 [file Table_1.docx]

**Table S1.** Multi-Locus Sequence Typing (MLST) allele combination, clonal complex to which STs are enclosed, associated strains and host.

| ST | *arcC* | *aroE* | *gtr* | *mutS* | *pyrR* | *tpiA* | *yqil* | CC | Strain | Host^a^ (no when >1) |
| --- | --- | --- | --- | --- | --- | --- | --- | --- | --- | --- |
| 2 | 7 | 1 | 2 | 2 | 4 | 1 | 1 | 5 | C3040 | H |
| 5 | 1 | 1 | 1 | 2 | 2 | 1 | 1 | 5 | C3922, C3044, C3046, C5110 | H (2), D (2) |
| 20 | 1 | 1 | 2 | 2 | 1 | 1 | 3 | 5 | C3933, C3934 | H (2) |
| 22 | 7 | 1 | 2 | 2 | 4 | 7 | 1 | 5 | C3910 | H |
| 35 | 2 | 1 | 2 | 2 | 4 | 1 | 1 | 5 | C3928 | H |
| 60 | 1 | 1 | 2 | 6 | 2 | 1 | 16 | 5 | C3036 | C |
| 83 | 1 | 2 | 1 | 2 | 1 | 1 | 10 | 5 | C3937 | H |
| 130 | 1 | 1 | 1 | 2 | 1 | 1 | 1 | 5 | C3033, PA84 | H, D (2) |
| 290 | 12 | 29 | 5 | 5 | 11 | 4 | 4 | 11 | C3034 | H |
| 553 | 7 | 1 | 48 | 2 | 4 | 7 | 1 | 5 | C3043 | H |
| 554 | 1 | 2 | 2 | 2 | 2 | 1 | 55 | 5 | C3926 | H |
| 555 | 60 | 2 | 2 | 2 | 2 | 1 | 3 | 5 | C3029, C3932 | H, D |
| 556 | 1 | 52 | 1 | 35 | 2 | 1 | 4 | - | C5112, C5114 | H (2) |
| 558 | 3 | 29 | 5 | 5 | 11 | 4 | 11 | 11 | C3035 | C |
| 559 | 12 | 44 | 5 | 5 | 3 | 4 | 20 | 11 | C3914 | H |
| 560 | 49 | 48 | 5 | 5 | 10 | 16 | 21 | 5- | C3921 | C |

^a^ H, human; D, dog; C, cat.

Novel STs are shown with a faint gray background, with novel alleles shown in dark grey background.
