# Supplementary material for: Clonally Diverse Methicillin and Multidrug Resistant Coagulase Negative Staphylococci Are Ubiquitous and Pose Transfer Ability Between Pets and Their Owners
Source: Front Microbiol. 2019 Mar 26;10:485. doi: 10.3389/fmicb.2019.00485 (PMC6443710; doi:10.3389/fmicb.2019.00485)
Supplement: Supplementary file 2 [file Table_2.docx]

**Table S2.** SCC*mec* and distribution *ccr* and *mec* complexes detected in the 31 investigated isolates.

| **House** | **Individual ID** | **Strain ID** | **Kondo *et al.,* 2007** | | | **Zhang *et al.,* 2005** | | **Consensus SCC*mec***^b^ |
| --- | --- | --- | --- | --- | --- | --- | --- | --- |
|  |  |  | ***ccr*** | ***mec* complex** | **Type**^a^ | **SCC*mec*** | **Type**^a^ |  |
| Households where at least one owner and one pet carried MRCoNS | | | | | | | | |
| **1** | **1-H1, 1-D1** | **C3031, C3030** | **2, 3** | **A** | **NA** | **-** | **NT** | **NT** |
|  | 1-H2, 1-D1 | C5116, C3029 | 2, C | C | NA | V | V | NA |
| **2** | **2-H1, 2-D1** | **C3041, C3033** | **2** | **B** | **IV** | **-** | **NT** | **NT** |
|  | 2-H2 | C3040 | - | A | NT | - | NT | NT |
| Households where only owners carried MRCoNS | | | | | | | | |
| 3 | 3-H1 | C3910 | - | A | NT | - | NT | NT |
| 4 | 4-H1 | C3034 | 2 | B | IV | IVa | IV | IV |
| 5 | 5-H1 | C5110 | 2 | B | IV | III, IVa | NA | NA |
| 6 | 6-H1 | C5112 | 2 | B | IV | - | NT | NT |
| 7 | 7-H1 | C3043 | - | A | NT | - | NT | NT |
| 8 | 8-H1 | C3914 | 2 | B | IV | IVa | IV | IV |
| 9 | 9-H1 | C5114 | 2 | B | IV | - | NT | NT |
| 10 | 10-H1 | C3922 | 2 | - | NT | III, IVa | NA | NT |
| 11 | 11-H1 | C3926 | C | C | V | V | V | V |
|  | 11-H2 | C3928 | 1, 2, C | A | NA | V | V | NA |
| 12 | 12-H1 | C3932 | 2, C | C | NA | V | V | NA |
|  | 12-H2 | C3933 | 1 | A | NA | - | NT | NT |
|  | 12-H3 | C3934 | 1, 2, 3 | A | NA | - | NT | NT |
| 13 | 13-H1 | C3937 | 2 | - | NT | IVa | IV | NT |
|  | 13-H2 | C3938 | 1, C | A | NA | V | V | NA |
| Households where only pets carried MRCoNS | | | | | | | | |
| 14 | 14-D1 | C3911 | 1 | A | NA | - | NT | NT |
| 15 | 15-D1 | C3913 | 1 | A | NA | - | NT | NT |
| 16 | 16-D1 | PA84 | 2 | B | IV | - | NT | NT |
| 17 | 17-D1 | C3044 | 2 | B | IV | III, IVa | NA | NA |
|  | 17-D2 | C3045 | 1, C | A | NA | III | III | NA |
|  | 17-D3 | C3046 | 2 | - | NT | III, IVa | NA | NT |
| 18 | 18-C1 | C3035 | 2, C | A | NA | I, V | NA | NA |
| 19 | 19-C1 | C3036 | - | - | NT | - | NT | NT |
| 20 | 20-C1 | C3921 | 2, C | B | NA | V | V | NA |

Data from cases of Interspecies Transmission and are marked in blot. SCC*mec* types concordant by both schemes as well as those identified by a single scheme are indicated in dark and faint gray, respectively.

^a^ NA, Non-Adscribed; NT, Non-Typeable.

^b^ Consensus SCC*mec* is determined by a combination of the type given by both schemes, prioritizing SCC*mec* NT, NA, and then the typeable cassettes.
