# Supplementary material for: Clonally Diverse Methicillin and Multidrug Resistant Coagulase Negative Staphylococci Are Ubiquitous and Pose Transfer Ability Between Pets and Their Owners
Source: Front Microbiol. 2019 Mar 26;10:485. doi: 10.3389/fmicb.2019.00485 (PMC6443710; doi:10.3389/fmicb.2019.00485)
Supplement: Supplementary file 3 [file Table_3.docx]

**Table S3**. Detailed information on all mutation positions detected in the quinolone resistance determining regions (QRDR) of GyrA (DNA Gyrase), ParC and ParE (DNA topoisomerase IV) of the quinolone resistant strains.

| Strain | *gyrA* | | | *gyrB* | *parC* | | | *parE* | | |
| --- | --- | --- | --- | --- | --- | --- | --- | --- | --- | --- |
|  | Codon | Nt^a^ pos | Amino acid involved |  | Codon | Nt pos | Amino acid involved | Codon | Nt pos | Amino acid involved |
| C3044, C3046 | CCG🡪CCT | 189 | P34P | WT^b^ | GTT🡪GTA | 210 | V70V | CTA🡪TTA | 1231 | L411L |
|  | GTG🡪GTC | 202 | V74V |  | CAG🡪CAA | 219 | Q73Q | TTA🡪CTA | 1288 | L430L |
|  | **TCT🡪TTT** | **251** | **S84F** |  | **TCT🡪TTT** | **239** | **S80F** | TTA🡪TTG | 1326 | L442L |
|  |  |  |  |  | **GAT🡪GGT** | **251** | **D84G** | GTG🡪GTT | 1374 | V458V |
|  |  |  |  |  | GGG🡪GGT | 312 | G104G |  |  |  |
| C3910 | CCG🡪CCT | 189 | P34P | WT | GTT🡪GTA | 210 | V70V | CTA🡪TTA | 1231 | L411L |
|  | GTG🡪GTC | 202 | V74V |  | CAG🡪CAA | 219 | Q73Q | TTA🡪CTA | 1288 | L430L |
|  | **TCT🡪TTT** | **251** | **S84F** |  | **TCT🡪TAT** | **239** | **S80Y** | TTA🡪TTG | 1326 | L442L |
|  | **GAA🡪AAA** | **262** | **E88K** |  | **GAT🡪TAT** | **250** | **D84Y** | GTG🡪GTT | 1374 | V458V |
|  |  |  |  |  | GGG🡪GGT | 312 | G104G |  |  |  |
| C3922 | CCG🡪CCT | 189 | P34P | WT | GTT🡪GTA | 210 | V70V | CTA🡪TTA | 1231 | L411L |
|  | GTG🡪GTC | 202 | V74V |  | CAG🡪CAA | 219 | Q73Q | TTA🡪CTA | 1288 | L430L |
|  | **TCT🡪TTT** | **251** | **S84F** |  | **TCT🡪TAT** | **239** | **S80Y** | TTA🡪TTG | 1326 | L442L |
|  | **GAA🡪AAA** | **262** | **E88K** |  | **GAT🡪TAT** | **250** | **D84Y** | GTG🡪GTT | 1374 | V458V |
|  |  |  |  |  | GGG🡪GGT | 312 | G104G |  |  |  |
| C5110 | CCG🡪CCT | 189 | P34P | WT | GTT🡪GTA | 210 | V70V | CTA🡪TTA | 1231 | L411L |
|  | GTG🡪GTC | 202 | V74V |  | CAG🡪CAA | 219 | Q73Q | TTA🡪CTA | 1288 | L430L |
|  | **TCT🡪TTT** | **251** | **S84F** |  | **TCT🡪TAT** | **239** | **S80Y** | TTA🡪TTG | 1326 | L442L |
|  |  |  |  |  | GGG🡪GGT | 312 | G104G | GTG🡪GTT | 1374 | V458V |

The substitutions implying amino acid change are indicated in bold.

^a^Nt, nucleotide

^b^WT, wild type
