# Supplementary material for: Clonally Diverse Methicillin and Multidrug Resistant Coagulase Negative Staphylococci Are Ubiquitous and Pose Transfer Ability Between Pets and Their Owners
Source: Front Microbiol. 2019 Mar 26;10:485. doi: 10.3389/fmicb.2019.00485 (PMC6443710; doi:10.3389/fmicb.2019.00485)
Supplement: Supplementary file 4 [file Table_4.docx]

**Table S4**. Prevalence (%) of MRCoNS and/or CoPS among tested and positive individuals.

|  | **Host (number)** | **MRCoNS and/or CoPS** | **One species** | | | | **More than one species** | | | | |
| --- | --- | --- | --- | --- | --- | --- | --- | --- | --- | --- | --- |
|  |  |  | **MRCoNS** | **CoPS** | **SA** | **SP** | | **SA + SP** | **MRCoNS + CoPS** | **MRCoNS + SA** | **MRCoNS + SP** |
| Tested individuals | **Owner** (68) | 55.88 | 11.74 | 27.94 | 26.47 | 1.47 | | 0.00 | 16.17 | 13.23 | 2.94 |
|  | **Pet** (66) | 45.5 | 10.61 | 28.79 | 6.06 | 21.21 | | 1.52 | 6.06 | 1.52 | 4.55 |
|  | \| **p-*value*** \| 0.2029 \| 1 \| 1 \| 0.0001686 \| 3,76E-03 \| 0.2386 \| 0.03998 \| 0.005451 \| 0.7209 \| \| --- \| --- \| --- \| --- \| --- \| --- \| --- \| --- \| --- \| --- \| | 0.203 | 1.000 | 1.000 | 1.686E-04 | 3.76E-03 | | 0.239 | 0.040 | 0.005 | 0.721 |
| Positive individuals | **Owner** (38) | - | 21.05 | 50.00 | 47.37 | 2.63 | | - | 31.58 | 23.68 | 5.26 |
|  | **Pet** (30) | - | 23.33 | 63.33 | 13.33 | 46.67 | | 3.33 | 13.33 | 3.33 | 10.00 |
|  | \| **p-*value*** \| 0,000 \| 0,865 \| 0,087 \| 0,000 \| 0,000 \| 0,246 \| 0,009 \| 0,014 \| 0,283 \| \| --- \| --- \| --- \| --- \| --- \| --- \| --- \| --- \| --- \| --- \| | - | 0.865 | 0.087 | 2.03E-04 | 6.22E-11 | | 0.246 | 0.002 | 0.014 | 0.283 |
