# Supplementary material for: Clonally Diverse Methicillin and Multidrug Resistant Coagulase Negative Staphylococci Are Ubiquitous and Pose Transfer Ability Between Pets and Their Owners
Source: Front Microbiol. 2019 Mar 26;10:485. doi: 10.3389/fmicb.2019.00485 (PMC6443710; doi:10.3389/fmicb.2019.00485)
Supplement: Supplementary file 6 [file Data_Sheet_1.docx]

Supplementary Material

**Clonally Diverse Methicillin and Multidrug Resistant Coagulase Negative Staphylococci are Ubiquitous and Pose Transfer Ability between Pets and their Owners**

Elena Gómez-Sanz^1,2*^, Sara Ceballos^3^, Laura Ruiz-Ripa^3^, Myriam Zarazaga^3^, Carmen Torres^3^

^1^ Institute of Food, Nutrition and Health, ETH Zurich, Zurich, Switzerland

^2^ Área de Microbiología Molecular, Centro de Investigación Biomédica de La Rioja (CIBIR), Logroño, Spain

^3^ Área Bioquímica y Biología Molecular, Universidad de La Rioja, Logroño, Spain

***Correspondence:** [elena.gomez@hest.ethz.ch](mailto:elena.gomez@hest.ethz.ch)

**Material and Methods**

**Isolation and identification of Coagulase Positive Staphylococci (CoPS): *Staphylococcus aureus* (SA) and *Staphylococcus pseudintermedius* (SP)**

Sampled nasal swabs were inoculated into Brain Heart Infusion broth (BHI; Difco) supplemented with 6.5% NaCl and incubated at 37 ◦C for 24 h. One-hundred microliters were inoculated on Oxacillin Resistant Staphylococcal Agar Base (ORSAB; OXOID) plates supplemented with 2 mg/L of oxacillin for the isolation of MRSA and MRSP. Seventy microliters were seeded on Manitol Salt Agar (MSA; BD) plates for the isolation of SA and SP. Plates were incubated at 37ºC for 24–48 h. Representative colonies were subcultured and further studied. Identification of isolates was based on colony morphology, Gram staining, and catalase and DNase activities. A multiplex PCR that amplifies the species specific *nuc* gene of SA or S*. intermedius* (SI)/SP was conducted to identify such species (Lautz et al., 2006). Discrimination between SI and SP was conducted by digestion of *pta* gene amplicon with MboI endonuclease (Bannoehr et al., 2009). The presence of *mecA* gene was investigated by PCR (Gomez-Sanz et al., 2011).

**Molecular typing of SA and SP isolates**

*Spa*-typing on SA isolates was performed following standard methodology (Gomez-Sanz et al., 2010) and sequences were analyzed using Ridom Staph-Type software version 1.5.21 (Ridom GmbH). Determination of the *agr* allotype of SA and SP was achieved by specific PCRs as previously described (Shopsin et al., 2003, Bannoehr et al., 2007). Multi Locus Sequence Typing (MLST) and subsequent assignment of clonal complexes (CC) was undergone as recommended (www.mlst.net; http://eburst.mlst.net) on SA isolates recovered in T0 (Gomez-Sanz et al., 2013a, Gomez-Sanz et al., 2013b).

Antimicrobial resistance profile, determination of cases of interspecies transmission and the longitudinal approach followed the same procedures as described in the manuscript.

**Results**

In case 1, an identical methicillin-susceptible SA (MSSA) strain was recovered from the same owner and dog (1-H1, 1-D1) that carried and identical multidrug resistant (MDR) methicillin-resistant *Staphylococcus lentus* (MRSL) clone in T0, representing a case of double interspecies transmission (IT), by both a CoPS and a methicillin-resistant coagulase negative (MRCoNS) clone. This owner revealed to be persistent carrier of the same MSSA clone whilst the dog was negative for the rest of the samplings (for in-depth analysis of this SA case along time please refer to Gomez-Sanz et al., (2013a). Interestingly, the same dog and owner 1-H2 concomitantly carried another MSSA clone in sampling T3, what represents another case of SA direct IT (Figure S1). In total, four different species (*S. aureus, S. lentus, Staphylococcus epidermidis* and *Staphylococcus haemolyticus*) were recovered from tested individuals from this household along the sampling year, with five different staphylococcal clones (3 MRCoNS and 2 MSSA). Of them, four (2 MRCoNS and 2 MSSA) were present in dog 1-D1.

In case 2, both owners carried an identical MSSA clone in T0 and T1, whilst this clone was replaced in successive samplings by non-related MSSA clones. Dog 2-D1 tested negative for CoPS along the whole sampling year (Figure S1). In total, two species (*S. aureus* and *S. epidermidis*) and 8 different clones (5 MRSE and 3 MSSA) were recovered from the three individuals tested. Of them, dog 2-D1 exhibited 3 different MRSE clones.

**References**

Bannoehr J., Ben Zakour N.L., Waller A.S., Guardabassi L., Thoday K.L., van den Broek A.H. and Fitzgerald J.R. (2007). Population genetic structure of the *Staphylococcus intermedius* group: insights into *agr* diversification and the emergence of methicillin-resistant strains. J Bacteriol 18923, 8685-8692.

Bannoehr J., Franco A., Iurescia M., Battisti A. and Fitzgerald J.R. (2009). Molecular diagnostic identification of *Staphylococcus pseudintermedius*. J Clin Microbiol 472, 469-471.

Gomez-Sanz E., Torres C., Ceballos S., Lozano C. and Zarazaga M. (2013a). Clonal dynamics of nasal *Staphylococcus aureus* and *Staphylococcus pseudintermedius* in dog-owning household members. Detection of MSSA ST(398). PLoS One 87, e69337.

Gomez-Sanz E., Torres C., Lozano C., Fernandez-Perez R., Aspiroz C., Ruiz-Larrea F. and Zarazaga M. (2010). Detection, molecular characterization, and clonal diversity of methicillin-resistant *Staphylococcus aureus* CC398 and CC97 in Spanish slaughter pigs of different age groups. Foodborne Pathog Dis 710, 1269-1277.

Gomez-Sanz E., Torres C., Lozano C., Saenz Y. and Zarazaga M. (2011). Detection and characterization of methicillin-resistant *Staphylococcus pseudintermedius* in healthy dogs in La Rioja, Spain. Comp Immunol Microbiol Infect Dis 345, 447-453.

Gomez-Sanz E., Torres C., Lozano C. and Zarazaga M. (2013b). High diversity of *Staphylococcus aureus* and *Staphylococcus pseudintermedius* lineages and toxigenic traits in healthy pet-owning household members. Underestimating normal household contact? Comp Immunol Microbiol Infect Dis 361, 83-94.

Lautz S., Kanbar T., Alber J., Lammler C., Weiss R., Prenger-Berninghoff E. and Zschock M. (2006). Dissemination of the gene encoding exfoliative toxin of *Staphylococcus intermedius* among strains isolated from dogs during routine microbiological diagnostics. J Vet Med B Infect Dis Vet Public Health 539, 434-438.

Shopsin B., Mathema B., Alcabes P., Said-Salim B., Lina G., Matsuka A., Martinez J. and Kreiswirth B.N. (2003). Prevalence of agr specificity groups among *Staphylococcus aureus* strains colonizing children and their guardians. J Clin Microbiol 411, 456-459.
