# Supplementary material for: Clonally Diverse Methicillin and Multidrug Resistant Coagulase Negative Staphylococci Are Ubiquitous and Pose Transfer Ability Between Pets and Their Owners
Source: Front Microbiol. 2019 Mar 26;10:485. doi: 10.3389/fmicb.2019.00485 (PMC6443710; doi:10.3389/fmicb.2019.00485)
Supplement: Supplementary file 7 [file Data_Sheet_2.docx]

**Figure S1**. Schematic representation of the CoPS and MRCoNS carriage dynamics of both households investigated along one year. IT, bacterial species responsible for interspecies transmission. T0 to T4 indicate the different sampling times along the sampling year. Individuals are named as follows: number of household (1-2), H (for human) or D (dog), and number (1 or 2) to distinguish individuals per household. The *spa* type and MLST in T0 of recovered CoPS isolates is shown on the right-hand side of the figure.
